# Association of muscle instability and long-term prophylaxis in hereditary angioedema^☆^

**DOI:** 10.1016/j.waojou.2026.101350

**Published:** 2026-02-26

**Authors:** Eleanor Hollers, Yunting Yu, James Sheetz, Kristina Richwine, Kara Grim, Rita Germak-Sovereign, Long Luong, Hirofumi Hitomi, Taha Al-Shaikhly, Timothy Craig

**Affiliations:** a Department of Medicine, Section of Allergy, Asthma & Immunology, Pennsylvania State University, Hershey, PA 17033, USA; b Thomas Jefferson University, Sidney Kimmel Medical College, Philadelphia, PA, 19107, USA; c Department of iPS Stem Cell & Regenerative Medicine, Kansai Medical University, Hirakata, Osaka, Japan

**Keywords:** Hereditary angioedema, Muscle, Prophylaxis, Fatigue, Creatine kinase, CPK

## Abstract

**Introduction:**

Hereditary angioedema (HAE) types 1 and 2 are caused by C1 inhibitor deficiency or dysfunction, leading to increased prekallikrein activity and bradykinin production. HAE causes vasodilation and edema resulting in obstruction of the upper airway, gastrointestinal symptoms, and skin swelling. Evidence of involvement of other organ systems has been sparse. Herein, we demonstrate evidence of creatinine kinase (CK) elevation in HAE patients suggesting an effect of bradykinin on skeletal muscle with subsequent improvement with long term prophylaxis (LTP).

**Methods:**

CK levels from participants with type 1 or 2 HAE enrolled in the Phase 2 and 3 clinical trials evaluating the safety and efficacy of donidalorsen for LTP in patients with HAE, was measured at baseline (before treatment initiation) and Week 17 (for participants enrolled in Phase 2 Study) and Week 25 (for participants enrolled in Phase 3 study). Mixed effect model with repeated measures was used to assess the influence of time and treatment (donidalorsen vs. placebo) on serum CK levels.

**Results:**

CK levels were available from 20 patients enrolled in the Phase 2 study and the mean CK level was numerically lower by Week 17; however, these results were not statistically significant. Among the 90 participants enrolled in the Phase 3 study who had CK levels checked at baseline and Week 25, a significantly lower CK level at Week 25 was observed among those receiving Q4W donidalorsen, but not among those receiving donidalorsen Q8W or placebo.

**Conclusion:**

Bradykinin appears to cause instability of skeletal muscle, causing CK release with even minor exercise. The effect of increases in bradykinin in HAE on muscle needs further research but may account for some of the atypical HAE symptoms patients often describe and which are noted in quality-of-life assessments. LTP, therefore, may confer additional benefits beyond reduction of HAE symptoms, potentially contributing to stabilization of skeletal muscle and improvement of fatigue and weakness.

## Introduction

Hereditary angioedema (HAE) type 1 (HAE-C1INH-Type1) and type 2 (HAE-C1INH-Type2), are autosomal dominant, inherited C1 esterase inhibitor (C1–INH) deficiency or dysfunction with inappropriate increases in bradykinin leading to acute nonpruritic swelling.^1^ C1–INH mediates the conversion of prekallikrein (PKK) to kallikrein via the inactivation of factor XIIa and inhibiting the activation of kallikrein directly.^2^ This inhibition of factor XII autoactivation and the resulting decrease in available kallikrein, which is needed to mediate the conversion of high-molecular-weight-kininogen (HMWK) to bradykinin, ultimately decreases the inflammatory bradykinin response.^2^ In those with HAE, this pathway is unchecked by C1–INH, leading to increased bradykinin and enhancement of its downstream effects including increased vascular permeability, as well as elevated levels of prostaglandin E2 (PGE2) and nitrous oxide (NO), and activation of the antifibrinolytic, complement, and coagulation pathways.^3^ These angioedema attacks often occur in the skin and submucosa of the gastrointestinal tract and upper respiratory tract, which can be fatal.^3^^,^^4^ Other symptoms can include weakness, fatigue, and decreased exercise tolerance which are often described by HAE patients and noted on quality of life surveys.^3^, ^4^, ^5^, ^6^

In addition to localized edema and submucosal swelling, bradykinin has been found to have a role in muscle injury and repair.^7^, ^8^, ^9^, ^10^ Bradykinin, acting through the kinin B2 receptor, induces prostaglandin E2 (PGE2) release from skeletal muscle derived fibroblasts, potentially increasing local inflammation and muscle damage.^7^ Regarding myocyte regeneration, blockade of the kinin-B2 receptor in murine myoblasts results in decreased skeletal muscle marker expression and decreased regenerative capacity due to ineffective cellular differentiation, indicated by the higher fraction of mononucleated myoblasts and reduced numbers of nascent myotubes observed in these models.^8^ During studies involving both strenuous weight bearing and aerobic exercise, elevated levels of bradykinin within the muscle tissue and may be implicated in delayed onset muscle soreness.^9^^,^^10^ These mechanisms suggest that elevated level of bradykinin at baseline and during attacks in HAE patients may predispose their skeletal muscle to increased PGE2, delayed onset muscle soreness, decreased regenerative ability, and increased muscle instability.

Traditionally, CK has been used as a marker of muscle pathology and damage due to its stability in serum assays, quick release from skeletal muscle cells after membrane disruption, and demonstrated tracking with strenuous muscle use.^11^^,^^12^ Additionally, CK is found in multiple isoenzyme forms depending on tissue origin, including the skeletal-muscle derived CK-MM.^13^ Several cases point to a possible link between CK and HAE, including a woman with myalgias and elevated CK in the presence of HAE as well as other cases of large CK-MM elevations in HAE patients despite minimal exercise.^14^^,^^15^

**Fig. 1:**
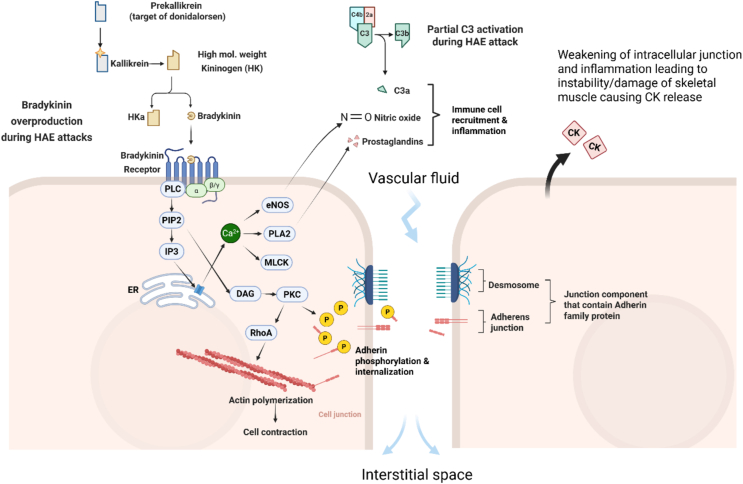
**Mechanisms of Bradykinin effect on intracellular junction stability and potentially induce muscle damage during HAE Attacks.** During HAE attacks, bradykinin is over-produced through the cleavage of high-molecular-weight kininogen (HK). It binds to bradykinin receptors and activates phospholipase C (PLC), which catalyzes the conversion of phosphatidylinositol bisphosphate (PIP2) into inositol trisphosphate (IP3) and diacylglycerol (DAG). IP3 stimulates the release of Ca2+ from the endoplasmic reticulum (ER), which then activates endothelial nitric oxide synthase (eNOS) and phospholipase A2 (PLA2), releasing nitric oxide (NO) and prostaglandins. Myosin light chain kinase (MLCK) and Ras homolog family member A (RhoA) promote actin polymerization, leading to cell contraction and widening of the interstitial space. DAG activates protein kinase C (PKC), which acts in the phosphorylation (through phosphate represented by yellow P) and internalization of adherens junction components (VE-cadherin in endothelial cells and M-cadherin in skeletal muscle cells). This, combined with increased immune cell recruitment and the inflammatory signaling of NO and prostaglandins, further weakens intercellular junctions and increases vascular permeability. The resulting structural instability and inflammation contribute to tissue damage and creatine kinase (CK) release from skeletal muscle

Long term prophylaxis (LTP) is often used to manage HAE and reduce attack frequency and severity. One investigational LTP option is donidalorsen, an antisense oligonucleotide that targets PKK mRNA, reducing the conversion to kallikrein and therefore bradykinin.^16^^,^^17^ The drug binds to PKK mRNA in the liver and leads to degradation, reducing its expression and translation (Fig. 1).^16^^,^^17^ Clinical trial data revealed a significant decrease in HAE attack frequency with a 4- and 8- week 80 mg dosing schedule showing better outcomes compared to placebo in terms of frequency and severity of attack while reducing the need for on-demand therapy.^17^^,^^18^ With this LTP therapy, we hypothesize that any muscle instability measured through elevations in serum CK will decrease along with disease severity and effective prophylaxis. Analysis of serum CK levels in HAE patients enrolled in trials of donidalorsen provides an opportunity to further investigate the relationship between the bradykinin production pathway and muscle instability.

## Methods

After signing IRB approved consents, patients with type 1 or 2 HAE who were enrolled in the Phase 2 and 3 clinical trials evaluating the efficacy and safety of donidalorsen, a selective inhibitior of plasma prekallikrein production, had serum CK levels measured at the time of enrollment before treatment initiation, and then at 1 time point after treatment initiation. Treatment included an 80 mg dose of donidalorsen or a placebo via injection every 4 (Q4W) or 8 (Q8W) weeks. For the Phase 2 study, CK was measured at Week 0 and Week 17. For the Phase 3 study, CK was measured at Week 0 and Week 25. Patients were blinded and protocol set by the clinical trial sponsor was followed. The clinical trial sponsor provided the blinded data and performed the statistical analyses based on a mixed effect model with repeated measures (MMRM) with fixed effects of treatment (donidalorsen or placebo), time (categorical), treatment-by-time interaction, and baseline value. A flowchart of full clinical trial methodology previously published with donidalorsen Phase II and III outcomes.^17^

## Results

**Fig. 2:**
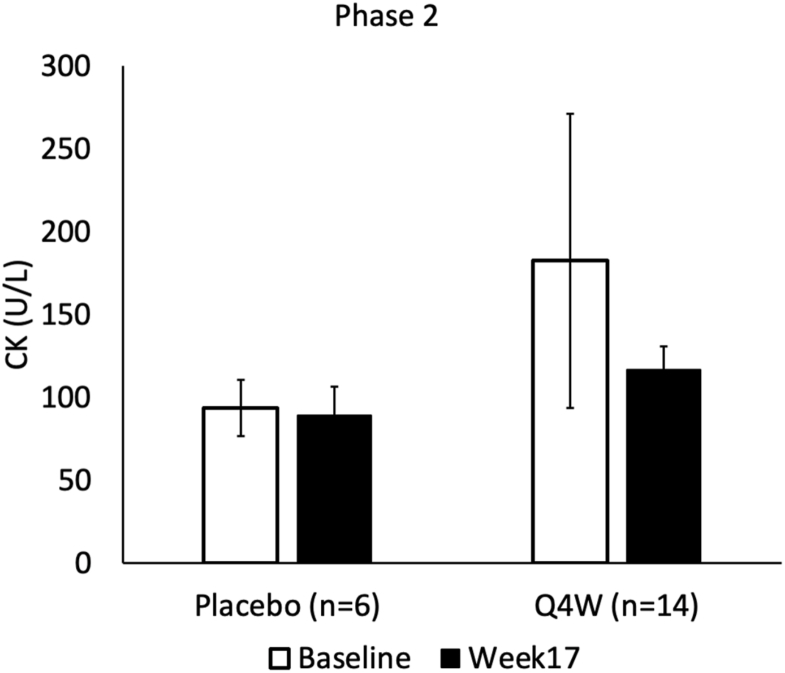
Creatine kinase (CK) measured in patients enrolled in the Phase 2 trial of donidalorsen receiving treatment (n = 14) or placebo (n = 6) every 4 weeks. A test based on a mixed effect model with repeated measures (MMRM) with fixed effects of treatment (donidalorsen or placebo), time (categorical), treatment-by-time interaction, and baseline value was performed to analyze the change in CK from initiation of treatment to 17 weeks after initiation (p = 0.299)

Twenty participants enrolled in the Phase 2 clinical trial (14 receiving donidalorsen treatment Q4W and 6 receiving placebo) had CK levels measured at baseline before treatment initiation and Week 17 post-treatment. Characteristics of the study participants were previously published, including that all participants save 1 were between 18 and 64 years of age, and that 64.7% were female.^19^ Mean serum CK level was numerically lower by Week 17 post-treatment compared to baseline only among those receiving treatment (Table 1, Fig. 2). Using a MMRM with fixed effects of treatment (donidalorsen or placebo), time (categorical), treatment-by-time interaction, and baseline value, CK level was not significantly different (p = 0.299). We attribute this to the small sample size (Table 1 and Fig. 2).

**Table 1:** Creatine kinase (CK) values as measured in patients enrolled in the Phase 2 trial of donidalorsen.

Phase 2	Placebo (n = 6)	Q4W (n = 14)
Baseline (mean ± SE)^a^	93.6 ± 16.9	182.5 ± 88.8
Week17 (mean ± SE)^a^	89.2 ± 17.3	116.8 ± 14.2
Change from baseline	−4.4 ± 14.6	−65.8 ± 92.1
p-value	n/a	0.299

a A mixed effect model with repeated measures (MMRM) with fixed effects of treatment (donidalorsen or placebo), time (categorical), treatment-by-time interaction, and baseline value was performed to analyze the change in CK (U/L) from initiation of treatment to Week 17 after initiation.

**Fig. 3:**
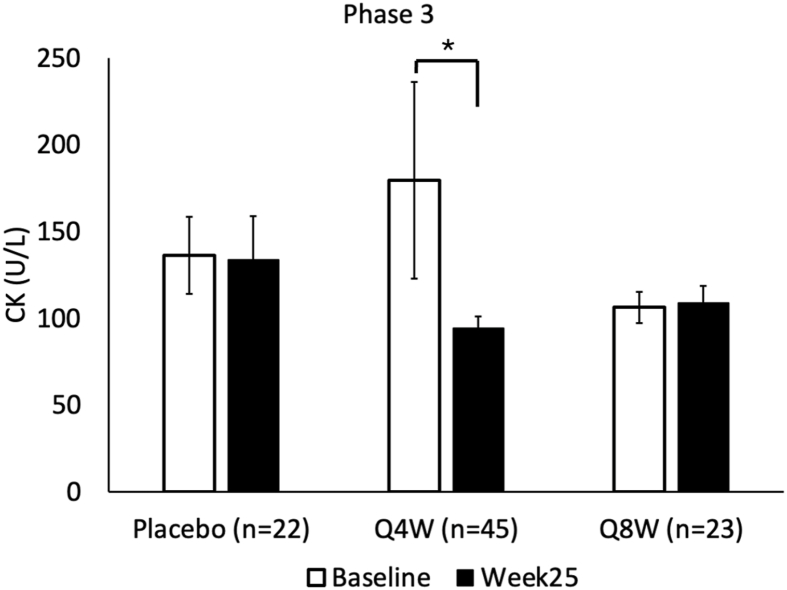
CK measured in patients enrolled in the Phase 3 trial of donidalorsen receiving treatment every 4 weeks (n = 45), 8 weeks (n = 23), or a placebo (n = 22). A test based on a mixed effect model with repeated measures (MMRM) with fixed effects of treatment (donidalorsen or placebo), time (categorical), treatment-by-time interaction, and baseline value was performed to analyze the change in CK in each condition independently (p < 0.05 for Q4W condition)

Using similar methodology, we analyzed serum CK levels from 90 study participants enrolled in the Phase 3 clinical trial, assessed both at baseline and Week 25 post-treatment (45 were receiving donidalorsen receiving treatment Q4W, 23 were receiving donidalorsen Q8W and 22 were receiving placebo). Characteristics of the study participants were previously published, including an age range of participants from 12 to 64 years of age except for 1, and 53% of participants were female.^17^ We observed a significant reduction in CK levels from baseline at Week 25 among participants receiving donidalorsen Q4W. Mixed effect analysis demonstrated a statistically significant difference from baseline (p = 0.016) among those receiving treatment Q4W (Table 2 and Fig. 3).

**Table 2:** CK measured in patients enrolled in the Phase 3 trial of donidalorsen.

Phase 3	Placebo (n = 22)	Q4W (n = 45)	Q8W (n = 23)
Baseline (mean ± SE)^a^	136.2 ± 22.1	179.5 ± 56.8	106.3 ± 9.1
Week25 (mean ± SE)^a^	133.8 ± 25.2	94.2 ± 6.8	108.7 ± 10.0
Change from baseline	2.8 ± 19.5	−88.5 ± 57.1	2.23 ± 8.6
p-value	n/a	0.016	0.286

a A mixed effect model with repeated measures (MMRM) with fixed effects of treatment (donidalorsen or placebo), time (categorical), treatment-by-time interaction, and baseline value was performed to analyze the change in CK (U/L) in each condition independently (p < 0.05 for treatment every 4 weeks, p > 0.05 for treatment every 8 weeks).

## Discussion

Prior research has demonstrated a link between bradykinin and muscle instability, however, the effect of HAE and chronic bradykinin exposure on skeletal muscle has not yet been explored. In patients with HAE, acute attacks may involve muscle degeneration along with the known local inflammation, potentially leading to a similar membrane instability and CK leakage as seen in strenuous exercise (mechanism proposed as seen in Fig. 1). If this mechanism is correct, elevated CK levels in these patients could function as an effective reflection of bradykinin-mediated muscle damage and edema seen in HAE.

Quality of life (QoL) surveys and other instruments demonstrate that activity impairment, fatigue, muscle weakness, and lowered exercise tolerance are common in patients with HAE, but no direct mechanism has been elicited.^5^^,^^20^, ^21^, ^22^ Treatment with LTP improves these symptoms and reduces HAE attacks.^22^ By comparing the serum level of CK in HAE patients before and after LTP with donidalorsen, we observed a significant decrease in CK serum levels over the course of treatment (Fig. 2, Fig. 3). Quality of life was assessed in parallel and showed improvement with donidalorsen treatment in domains including fatigue, weakness, and exercise tolerance.^17^^,^^23^ This response in our marker (CK) for muscle instability suggests a chronic bradykinin effect on skeletal muscle in HAE patients that can be mitigated via the use of LTP. Additionally, our findings suggest that there could be an additional benefit to using LTP through stabilizing muscle, and might serve to improve myalgias, fatigue, and increasing exercise tolerance in addition to decreasing HAE attacks and severity, as observed in these studies.^17^^,^^23^

Potential limitations to our study include our small sample sizes of patients enrolled in the Phase II and Phase III donidalorsen trials for whom CK serum levels were collected. The small sample size, however, is consistent with other HAE studies due to the rarity of the disease. Additionally, we lack longitudinal data across the weeks of treatment to see if the impact on CK is continuous and whether there is an increase in exercise or improvement of fatigue associated with the improvement of CK. As CK level collection in this study and our subsequent findings were incidental, we acknowledge the lack of data on patient exercise and fitness level which may impact CK levels. We also acknowledge the lack of median analysis and hope to pursue a broader data collection and analysis in the future.

Our findings suggest that if CK truly correlates with disease severity, along with symptoms of fatigue and myalgias, and if it correlates with response to LTP, it could be an important tool in the follow-up and treatment of HAE patients on LTP (although not for C1–INH LTP for which C1-functional levels can be assessed). These data also suggest that in HAE patients with significant fatigue, activity impairment and exercise intolerance, a trial of LTP may be indicated.

Further research is needed to explore the pathologic changes occurring in the muscle tissue in association with poor control of the contact and complement pathways, whether there is a potential association between QoL measures, symptoms of fatigue, and exercise limitation with serum CK levels in patients with HAE. Researching these questions could shed light into whether symptoms such as fatigue and exercise limitation could be an indication to treat HAE irrespective of HAE attack burden.

## Abbreviations

HAE, Hereditary angioedema; CK, Creatine kinase; PKK, Prekallikrein; QoL, Quality of Life; LTP, Long term prophylaxis; Q4W, Dosing every 4 weeks; Q8W, Dosing every 8 weeks; PGE2, Prostaglandin E2; NO, Nitrous oxide; C1–INH, C1 esterase inhibitor; MMRM, Mixed effect model with repeated measures

## Availability of data and materials

Data supporting this study cannot be shared due to agreements with Ionis Pharmaceuticals. Data supplied by Ionis Pharmaceuticals, Carlsbad, CA, 92,010.

## Author contributions

EH, YY, and TC conceived of the study. TC and TA supervised the findings and guided analysis. LL and HH created figures and provided expertise. KR, KG, and RG enrolled patients and managed consent and IRB approval. EH, YY, JS, TA, and TC wrote the manuscript with input from all authors.

## Ethics approval

**Penn State Institutional Review Board**.

**Phase 2**.

IRB#13418-Phase 2: ISIS 721744-CS2-A Randomized, Double-Blind, Placebo-Controlled, Phase 2a Study to Assess the Clinical Efficacy of ISIS 721744, a Second-Generation Ligand-Conjugated Antisense Inhibitor of Prekallikrein, in Patients with Hereditary Angioedema-**IRB Approval: 2/21/2020**.

IRB#15367-Phase 2: ISIS 721744-CS3-An Open-Label Extension Study of ISIS 721744 in Patients with Hereditary Angioedema-**IRB Approval: 7/15/2020---** this was the open label extension study to ISIS 721744-CS2.

**Phase 3**.

IRB #19558-Phase 3: ISIS 721744-CS5-A Phase 3 Double-Blind, Placebo-Controlled Study to Evaluate the Efficacy and Safety of ISIS 721744 in Patients with Hereditary Angioedema (HAE)-**IRB Approval: 1/22/2022**.

IRB #20621-Phase 3: ISIS 721744-CS7-An Open-Label, Long Term Safety and Efficacy Study of Donidalorsen in the Prophylactic Treatment of Hereditary Angioedema (HAE)-**IRB Approval: 11/17/2022**---this was the open label extension study to ISIS 721744-CS5.

## Authors' consent for publication

All authors have read and approved of this manuscript for publication.

## Disclosure statement regarding use of generative artificial intelligence (AI) and AI-assisted technologies

Nothing to disclose.

## Funding

None.

## Conflict of interest

TC is the only author with conflicts of interest. His conflicts are: T.J. Craig is a speaker for CSL Behring, Grifols, KalVista Pharmaceuticals, and Takeda Pharmaceuticals; has received research and consultancy grants from Astria Therapeutics, BioCryst, BioMarin Pharmaceutical, CSL Behring, Grifols, ARADx, Ionis Pharmaceuticals, KalVista Pharmaceuticals, Pharvaris, and Takeda Pharmaceuticals; and is on the Medical Advisory Board for the US Hereditary Angioedema Association, Director of ACARE Angioedema Center at Penn State University, Hershey, PA, USA.

## References

[1] William R., Lumry M.D. (2013 Jun 25). https://www.ajmc.com/view/ace010_13jun_lumry1_s103to10.

[2] Henry Li H., Riedl M., Kashkin J. (2019 Apr 1). Update on the use of C1-Esterase inhibitor replacement therapy in the acute and prophylactic treatment of hereditary angioedema. Clin Rev Allergy Immunol.

[3] Kaplan A.P., Joseph K. (2010 Mar 1). The bradykinin-forming cascade and its role in hereditary angioedema. Ann Allergy Asthma Immunol.

[4] Bork K., Ressel N. (2003 Dec 1). Sudden upper airway obstruction in patients with hereditary angioedema. Transfus Apher Sci.

[5] Mak HWF, Wong JCY, Chiang V, Lam DLY, Li PH. From anxiety to work productivity and activity impairment: the mediating role of fatigue in hereditary angioedema. Clin Exp Allergy [Internet]. [cited 2025 May 11] https://onlinelibrary.wiley.com/doi/abs/10.1111/cea.14632. 10.1111/cea.14632 PMC12127057 39861951

[6] Weller K., Magerl M., Peveling-Oberhag A., Martus P., Staubach P., Maurer M. (2016 Aug). The angioedema quality of life questionnaire (AE-QoL) - assessment of sensitivity to change and minimal clinically important difference. Allergy.

[7] Muscella A., Cossa L.G., Vetrugno C., Marsigliante S. (2020 May 1). Bradykinin stimulates prostaglandin E2 release in human skeletal muscular fibroblasts. Mol Cell Endocrinol.

[8] Alves J.M., Martins A.H., Lameu C. (2019 Feb 1). Kinin-B2 receptor activity in skeletal muscle regeneration and myoblast differentiation. Stem Cell Rev Rep.

[9] Blais C., Adam A., Massicotte D., Péronnet F. (1999 Sep). Increase in blood bradykinin concentration after eccentric weight-training exercise in men. J Appl Physiol.

[10] Langberg H., Bjørn C., Boushel R., Hellsten Y., Kjaer M. (2002 Aug 1). Exercise-induced increase in interstitial bradykinin and adenosine concentrations in skeletal muscle and peritendinous tissue in humans. J Physiol.

[11] McLeish M.J., Kenyon G.L. (2005 Jan 1). Relating structure to mechanism in creatine kinase. Crit Rev Biochem Mol Biol.

[12] Brancaccio P., Maffulli N., Limongelli F.M. (2007 Jan 1). Creatine kinase monitoring in sport medicine. Br Med Bull.

[13] Takagi Y., Yasuhara T., Gomi K. (2001 Nov 1). [Creatine kinase and its isozymes]. Rinsho Byori.

[14] Beck G., Yamashita R., Saeki C., Ogawa T., Shimizu M., Mochizuki H. (2020). C1-inhibitor deficiency induces myositis-like symptoms via the deposition of the membrane attack complex in the muscle. Intern Med.

[15] Yu Y., Hollers E., Luong L. (2024 Nov 1). Muscle instability associated with hereditary angioedema. Ann Allergy Asthma Immunol.

[16] Smith T.D., Riedl M.A. (2024 Oct 1). The future of therapeutic options for hereditary angioedema. Ann Allergy Asthma Immunol.

[17] Riedl M.A., Tachdjian R., Lumry W.R. (2024 Jul 3). Efficacy and safety of donidalorsen for hereditary angioedema. N Engl J Med.

[18] Raja A., Shuja M.H., Raja S. (2024 Dec 12). Efficacy and safety of donidalorsen in hereditary angioedema with C1 inhibitor deficiency: a systematic review and a meta-analysis. Arch Dermatol Res.

[19] Petersen R.S., Bordone L., Riedl M.A. (2024). A phase 2 open-label extension study of prekallikrein inhibition with donidalorsen for hereditary angioedema. Allergy.

[20] Zarnowski J., Treudler R. (2024 Nov 14). Dietary and physical trigger factors in hereditary angioedema: self-conducted investigation and literature overview. Allergol Select.

[21] Prior N., Remor E., Pérez-Fernández E. (2016). Psychometric field study of hereditary angioedema quality of life questionnaire for adults: hae-qol. J Allergy Clin Immunol Pract.

[22] Banerji A., Davis K.H., Brown T.M. (2020 Jun). Patient-reported burden of hereditary angioedema: findings from a patient survey in the United States. Ann Allergy Asthma Immunol.

[23] Cohn D., Yarlas A., Bjorner J. (2025 Feb 1). Improvements in quality-of-life in patients with HAE receiving donidalorsen: post hoc analysis from the OASIS-HAE study. J Allergy Clin Immunol.

